# Sphingosine kinase 1/sphingosine-1-phosphate (S1P)/S1P receptor axis is involved in ovarian cancer angiogenesis

**DOI:** 10.18632/oncotarget.20471

**Published:** 2017-08-24

**Authors:** Lan Dai, Yixuan Liu, Lei Xie, Xia Wu, Lihua Qiu, Wen Di

**Affiliations:** ^1^ Department of Obstetrics and Gynecology, Ren Ji Hospital, School of Medicine, Shanghai Jiao Tong University, Shanghai 200127, China; ^2^ Shanghai Key Laboratory of Gynecologic Oncology, Ren Ji Hospital, School of Medicine, Shanghai Jiao Tong University, Shanghai 200127, China; ^3^ State Key Laboratory of Oncogene and Related Genes, Shanghai Cancer Institute, Ren Ji Hospital, School of Medicine, Shanghai Jiao Tong University, Shanghai 200127, China

**Keywords:** sphingosine kinase 1 (SphK1), sphingosine-1-phosphate (S1P), S1P receptor (S1PR), angiogenesis, ovarian cancer

## Abstract

Sphingosine kinase (SphK)/sphingosine-1-phosphate (S1P)/S1P receptor (S1PR) signaling pathway has been implicated in a variety of pathological processes of ovarian cancer. However, the function of this axis in ovarian cancer angiogenesis remains incompletely defined. Here we provided the first evidence that SphK1/S1P/S1PR_1/3_ pathway played key roles in ovarian cancer angiogenesis. The expression level of SphK1, but not SphK2, was closely correlated with the microvascular density (MVD) of ovarian cancer tissue. *In vitro*, the angiogenic potential and angiogenic factor secretion of ovarian cancer cells could be attenuated by SphK1, but not SphK2, blockage and were restored by the addition of S1P. Moreover, in these cells, we found S1P stimulation induced the angiogenic factor secretion via S1PR_1_ and S1PR_3_, but not S1PR_2_. Furthermore, inhibition of S1PR_1/3_, but not S1PR_2_, attenuated the angiogenic potential and angiogenic factor secretion of the cells. *in vivo*, blockage of SphK or S1PR_1/3_ could attenuate ovarian cancer angiogenesis and inhibit angiogenic factor expression in mouse models. Collectively, the current study showed a novel role of SphK1/S1P/S1PR_1/3_ axis within the ovarian cancer, suggesting a new target to block ovarian cancer angiogenesis.

## INTRODUCTION

Angiogenesis, the process through which new blood vessels sprout from pre-existing ones, is one of the hallmarks of cancer [1]. Recently, abundant evidence has indicated that ovarian cancer progression has close interactions with angiogenesis [2–5]. Indeed, angiogenesis is induced early and remained on during the multistage development of ovarian cancer, providing nutrients and oxygen for sustained neoplastic growth [2]. In turn, ovarian cancer cells have also been shown to be essential for regulating tumor-associated angiogenesis by secreting a series of proangiogenic growth factors into the tumor microenvironment [3]. Besides, increased angiogenesis was associated with increased risk of disease progression in the patients with advanced-stage epithelial ovarian cancer (EOC) [4]. Moreover, anti-angiogenic therapy with Bevacizumab, a humanized recombinant antibody against vascular endothelial growth factor (VEGF), revealed a more pronounced progression-free survival in the patients with advanced EOC [5]. However, the current understanding of molecular mechanisms of ovarian cancer angiogenesis remains incomplete.

Sphingosine kinase (SphK), the key enzyme to produce sphingosine-1-phosphate (S1P), exhibits two isoforms, SphK1 and SphK2. SphK1 has emerged as a significant signaling enzyme because it contributes to the growth, metastasis and chemoresistance of various human cancers [6, 7]. The “inside-out” model is widely used to explain the function of SphK1/S1P/S1P receptor (S1PR) signaling, which propose that intracellular S1P, generated by SphK, is secreted into the extracellular milieu and then activates cell surface S1PRs through autocrine and paracrine manners, leading to the activation of downstream signals [8]. However, the function of SphK2 has been less investigated and its roles and mechanisms in cancer remain largely unknown. In our previous study, we found that the level of SphK1 was significantly increased in ovarian cancer tissues [9]. Besides, earlier studies showed that the plasma S1P level was elevated in patients with ovarian cancer and decreased after tumor removal surgery [10]. Moreover, S1P has been shown to be involved in vascular tube formation process, including vascular maturation [11] and permeability [12]. Therefore, we hypothesized that SphK/S1P/S1PR signaling pathway may have an important influence on the angiogenic process of ovarian cancer.

Based on the above scientific background, our aim was to reveal the roles of SphK/S1P/S1PR pathway in ovarian cancer angiogenesis. The current study was designed to evaluate the correlation between SphK expression level and the microvascular density of ovarian cancer tissue, and to investigate the *in vitro* and *in vivo* effects of targeting SphK or S1PRs with specific antagonists or siRNAs on ovarian cancer angiogenesis.

## RESULTS

### Elevated levels of SphK1 was accompanied by increased microvascular density (MVD) in ovarian cancer tissue

**Figure 1:**
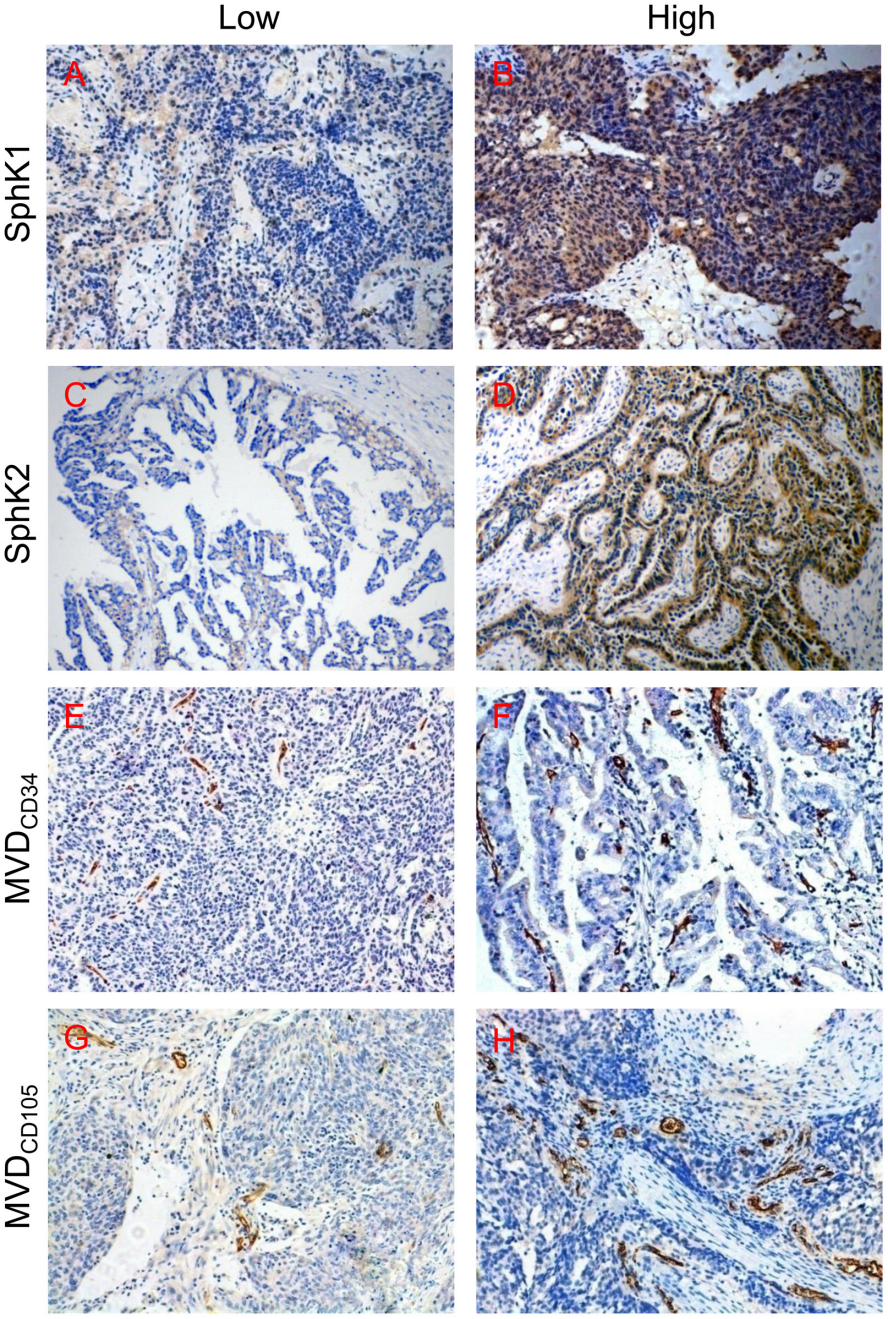
Immunohistochemical staining of SphK1, SphK2, CD34 and CD105 in epithelial ovarian cancer tissues **(A)** Low SphK1 expression; **(B)** high SphK1 expression; **(C)** low SphK2 expression; **(D)** high SphK2 expression; **(E)** low MVD_CD34_ area; **(F)** high MVD_CD34_ area; **(G)** low MVD_CD105_ area; **(H)** high MVD_CD105_ area. Magnification, 200× (A–H).

We investigated the SphK1, SphK2, MVD_CD34_ and MVD_CD105_ expression in samples from ovarian cancer patients. Figure 1A, 1C, 1E and 1G showed the representative fields of low SphK1, SphK2, MVD_CD34_ and MVD_CD105_ in these samples respectively. For comparison, Figure 1B, 1D, 1F and 1H showed the representative fields of high SphK1, SphK2, MVD_CD34_ and MVD_CD105_ respectively. In the current study, we found both MVD_CD34_ and MVD_CD105_ were significantly correlated with the levels of SphK1 in tumor tissue (Table 1). However, neither MVD_CD34_ nor MVD_CD105_ were correlated with the expression levels of SphK2. These findings suggested that SphK1 might be related to ovarian cancer angiogenesis.

**Table 1:** Spearman correlation analysis of SphK1/2 expression and microvascular density (MVD)

		SphK1		Correlation coefficients (ρ)	P value
		Low (n=23)	High (n=27)		
MVD_CD34_	Low (n=28)	20	8	0.576	<0.01
	High (n=22)	3	19		
		**SphK1 **		**Correlation coefficients (ρ)**	**P value**
		**Low (n=23)**	**High (n=27)**		
MVD_CD105_	Low (n=28)	19	9	0.495	<0.01
	High (n=22)	4	18		
		**SphK2 **		**Correlation coefficients (ρ)**	**P value**
		**Low (n=14)**	**High (n=36)**		
MVD_CD34_	Low (n=28)	7	21	-0.075	0.603
	High (n=22)	7	15		
		**SphK2 **		**Correlation coefficients (ρ)**	**P value**
		**Low (n=14)**	**High (n=36)**		
MVD_CD105_	Low (n=28)	6	22	-0.165	0.252
	High (n=22)	8	14		

### Inhibition of SphK by SKI-II suppressed the angiogenic potential and inhibited S1P and angiogenic factor secretion of ovarian cancer cells

**Figure 2:**
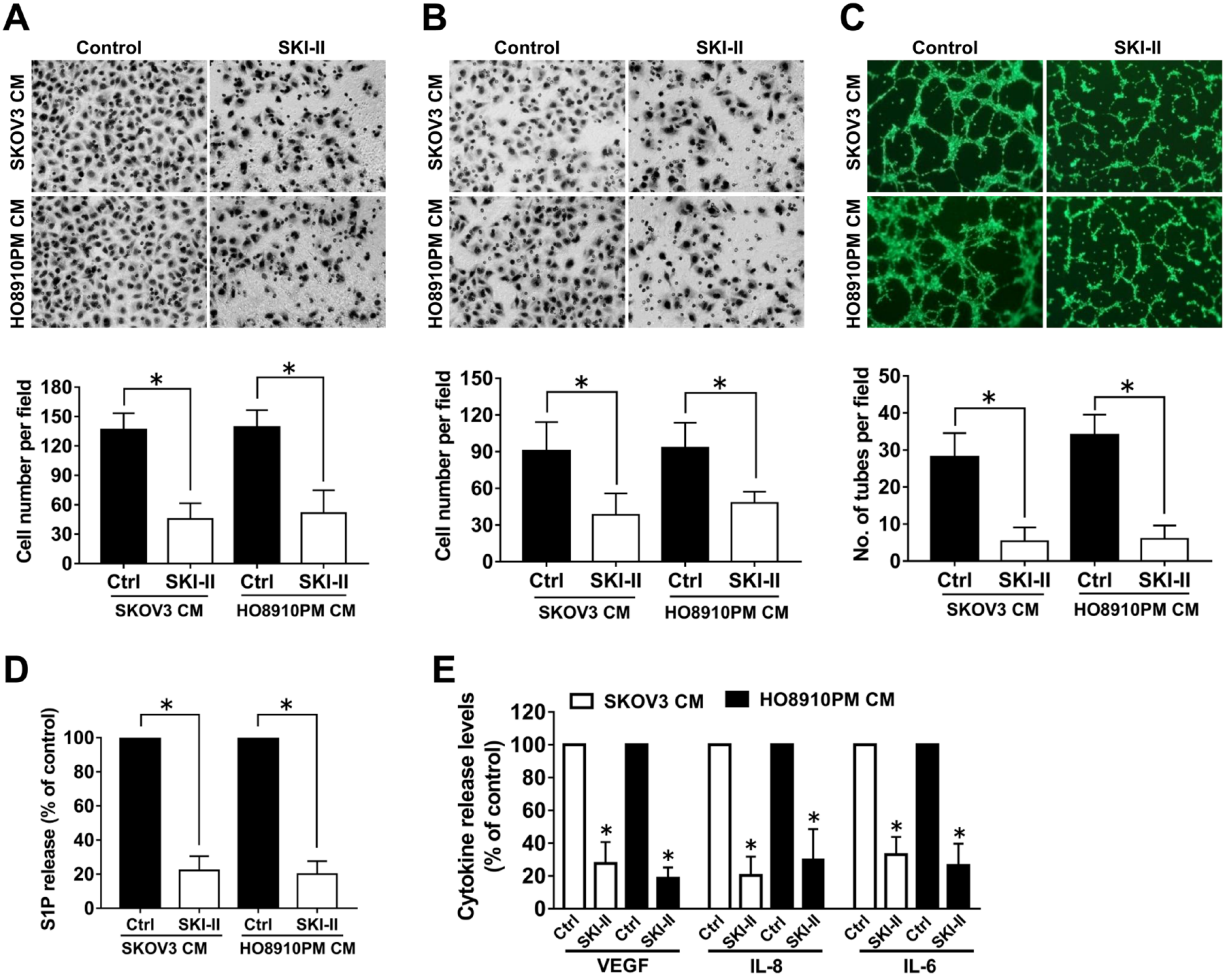
Effect of SphK inhibition by SKI-II on angiogenesis *in vitro* **(A)** Cell migration assay. Endothelial cells were stimulated with the culture media (CM) collected from the ovarian cancer cells precultured with or without SKI-II (2.5μM). The migrated cells were stained, photographed and counted. **(B)** Cell invasion assay. Endothelial cells were stimulated with CM, the invaded cells were photographed and counted. **(C)** Tube formation assay. Endothelial cells suspended in CM were placed on the matrigel to form tube like structures, which were then photographed and quantificated. **(D)** Effect of SKI-II on the S1P secretion of ovarian cancer cells. **(E)** Effect of SKI-II on the VEGF, IL-8 and IL-6 secretion of ovarian cancer cells. All experiments were repeated three times, with three replicates in each group (* p<0.05 vs. Control group).

In order to further determine whether SphK was involved in ovarian cancer angiogenesis, we examined the effects of SphK inhibition by SKI-II, a highly SphK selective inhibitor, on the angiogenic potential of ovarian cancer cells through using endothelial cell migration, invasion and tube formation assays. We found the culture medium prepared from SKI-II pretreated ovarian cancer cells significantly inhibited the migration, invasion and tube formation ability of endothelial cells (Figure 2A-2C), which suggested that SphK might play an important role in ovarian cancer angiogenesis. It is known that SphK activation leads to S1P formation, which in turn regulates a variety of cellular functions. To evaluate the role of S1P in SphK-induced ovarian cancer angiogenesis, we measured the S1P secretion levels in the supernant of ovarian cancer cells after SphK inhibition. As expected, SphK blockage suppressed the S1P secretion by ovarian cancer cells. Moreover, VEGF, interleukin-8 (IL-8) and interleukin-6– (IL-6) have been identified to play important roles in ovarian cancer angiogenesis [13–15]. We next examined the secretion levels of these angiogenic factors after SKI-II pretreatment. As shown in Figure 2E, SphK blockage also suppressed VEGF, IL-8 and IL-6 secretion of the cancer cells. Taken together, these data suggested that SphK blockage could attenuate the *in vitro* angiogenic potential and inhibit S1P and angiogenic factor secretion of ovarian cancer cells.

### SphK1, but not SphK2, was responsible for the angiogenic potential of ovarian cancer cells

**Figure 3:**
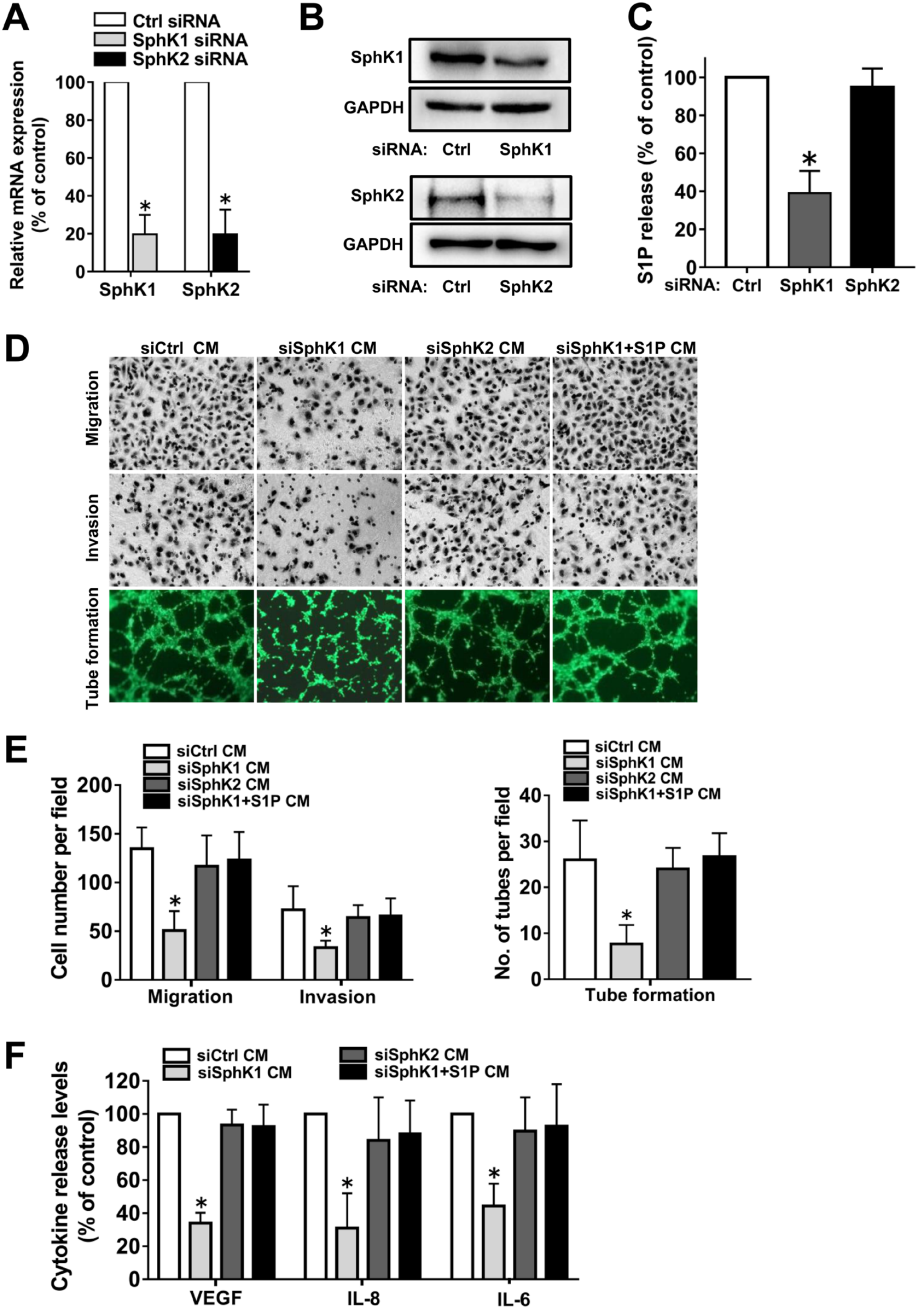
Effect of SphK1 or SphK2 blockage on angiogenesis *in vitro* **(A)** SKOV3 cells were transfected with control-siRNA, SphK1-siRNA or SphK2-siRNA. Expression of SphK1 or SphK2 mRNA levels were determined by PCR and normalized to GAPDH mRNA. **(B)** Protein levels of SphK1 and SphK2 were determined by western blot. **(C)** S1P levels in the culture media (CM) from siRNA transfected cells were determined by ELISA. **(D)** Representative images of the migration, invasion and tube formation assays. Endothelial cells were stimulated with the CM collected from siRNA transfected cells with or without S1P (1μM) addition. Migrated cells, invaded cells and tube like structures were photographed. **(E)** Statistical analysis of the migrated cells, invaded cells and tube like structures. **(F)** Effect of siRNA transfection with or without S1P addition on the VEGF, IL-8 and IL-6 secretion of ovarian cancer cells. All experiments were repeated three times, with three replicates in each group (* p<0.05 vs. Control group).

Having shown that SphK inhibition by SKI-II attenuated the angiogenic potential of ovarian cancer cells, we aimed to determine which isoform was involved in this process. For this purpose, specific siRNAs were used to down-regulate the expression levels of SphK1 or SphK2 (Figure 3A-3B). SphK1, but not SphK2, blockage significantly decreased S1P release from ovarian cancer cells (Figure 3C). Moreover, SphK1, but not SphK2, siRNA transfection significantly attenuated the angiogenic potential and angiogenic factor secretion of ovarian cancer cells (Figure 3D-3F), which suggested that SphK1, but not SphK2, was involved in ovarian cancer angiogenesis. Furthermore, these changes of phenotypes induced by SphK1 blockage could be reversed by exogenous S1P addition (Figure 3D-3F), which suggested that SphK1 blockage attenuated the angiogenic potential of ovarian cancer cells partly through inhibition of S1P.

### S1PR_1_ and S1PR_3_ mediated S1P-induced angiogenic factor secretion in ovarian cancer cells

**Figure 4:**
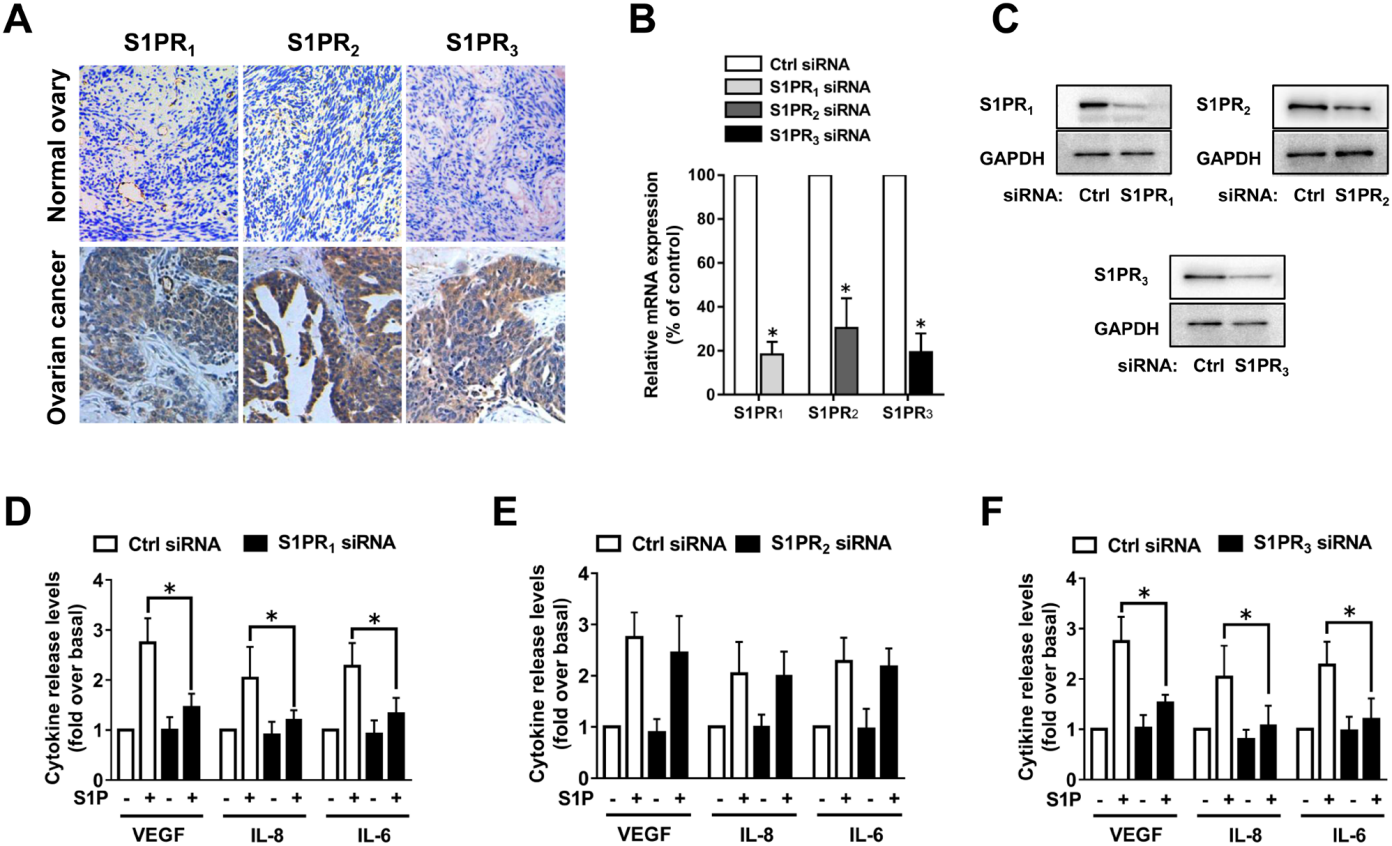
S1PR1 and S1PR3 mediate S1P-induced VEGF, IL-8 and IL-6 expression **(A)** Immunohistochemical staining of S1PR_1_, S1PR_2_ and S1PR_3_ in human normal ovarian tissue and ovarian cancer tissue. **(B)** SKOV3 cells were transfected with control-siRNA, S1PR_1_-siRNA, S1PR_2_-siRNA or S1PR_3_-siRNA. Expression levels of S1PR_1_, S1PR_2_ and S1PR_3_ were determined by PCR and normalized to GAPDH mRNA. **(C)** S1PR_1_, S1PR_2_ and S1PR_3_ proteins were determined by western blot. **(D)** Effects of S1PR_1_-siRNA on VEGF, IL-8 and IL-6 secretion in response to S1P treatment. **(E)** Effects of S1PR_2_-siRNA on VEGF, IL-8 and IL-6 secretion in response to S1P treatment. **(F)** Effects of S1PR_3_-siRNA on VEGF, IL-8 and IL-6 secretion in response to S1P treatment. All experiments were repeated three times, with three replicates in each group (* p<0.05 vs. Control group).

SphK blockage suppressed VEGF, IL-8 and IL-6 secretion in ovarian cancer cells (Figures 2E and 3F). As the roles of SphK mainly depend on its production of S1P, we investigated whether S1P could promote the angiogenic factor secretion. We found that S1P could induce substantial increases of VEGF, IL-8 and IL-6 release from ovarian cells *in vitro* (data not shown). S1P can function either intracellularly or extracellularly, such as through S1P receptors. Since S1PR_1-3_ are widely expressed in almost all types of cells, we examined S1PR_1-3_ expression levels in 20 ovarian cancer samples and 10 normal ovarian tissue samples, as shown in Figure 4A. In ovarian cancer, we observed that 55% (11/20) samples displayed high S1PR_1_ level, 80% (16/20) samples showed high S1PR_2_ level and 75% (15/20) samples showed high S1PR_3_ level. In contrast, in normal ovarian tissue, we observed 10% (1/10) samples displayed high S1PR_1_ level, 20% (2/10) samples showed high S1PR_2_ level and 20% (2/10) samples showed high S1PR_3_ level. Then we investigated which subtypes of S1PRs were implicated in the stimulation of VEGF, IL-8 and IL-6 by S1P by using siRNAs (Figure 4B-4C). As shown in Figure 4D-4F, knockdown of S1PR_1_ or S1PR_3_ significantly abrogated the secretion of VEGF, IL-8 and IL-6 induced by S1P. In contrast, S1PR_2_ siRNA did not alter the effects of S1P on VEGF, IL-8 and IL-6 secretion. These results suggested that S1PR_1_ and S1PR_3_ might be involved in S1P-induced VEGF, IL-8 and IL-6 secretion in ovarian cancer cells, while S1PR_2_ had almost no effect on the process.

### S1PR_1/3_ was responsible for the angiogenic potential of ovarian cancer cells

**Figure 5:**
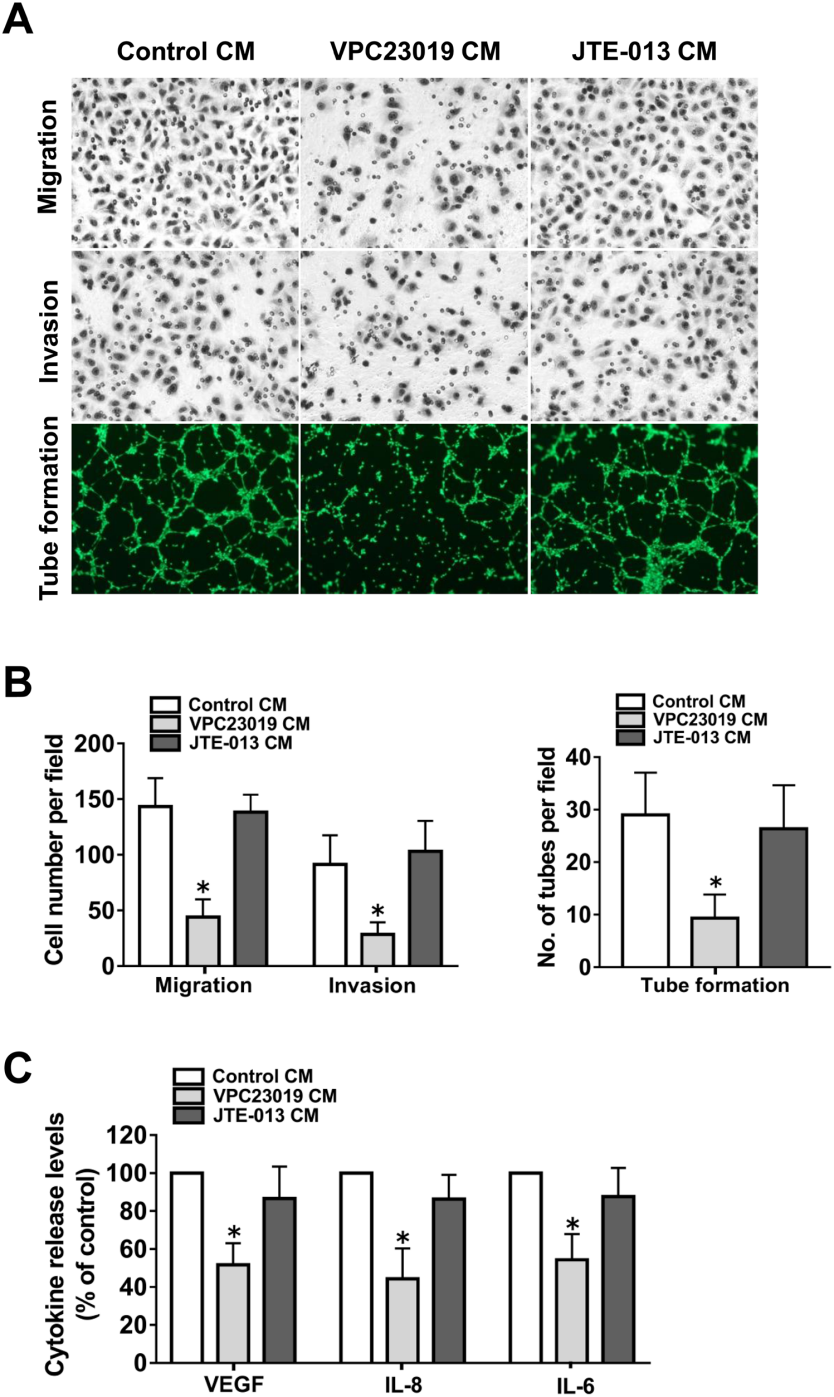
Effect of S1PR1-3 inhibition on angiogenesis *in vitro* **(A)** Representative images of the migration, invasion and tube formation assays. Endothelial cells were stimulated with CM collected from the ovarian cancer cells precultured with VPC23019 (300nM) or JTE-013 (1μM). Migrated cells, invaded cells and tube like structures were photographed. **(B)** Statistical analysis of the migrated cells, invaded cells and tube like structures. **(C)** Effect of VPC23019 and JTE-013 on the VEGF, IL-8 and IL-6 secretion of ovarian cancer cells. All experiments were repeated three times, with three replicates in each group (* p<0.05 vs. Control group).

Because S1PR_1_ and S1PR_3_ mediated S1P-induced angiogenic factor secretion in ovarian cancer cells, we wanted to determine whether they are responsible for the angiogenic potential of the cells. To this end, specific S1PRs antagonists were used. As shown in Figure 5A-5B, the culture medium prepared from S1PR_1/3_ antagonist, VPC23019, pretreated ovarian cancer cells significantly inhibited endothelial cell migration, invasion and tube formation. We next examined VEGF, IL-8 and IL-6 secretion levels after VPC23019 pretreatment. As shown in Figure 5C, S1PR_1/3_ blockage suppressed the angiogenic factor secretion of ovarian cancer cells. In contrast, S1PR_2_ antagonist, JTE-013, pretreatment had almost no effect on the angiogenic potential and the angiogenic factor secretion of the cancer cells. The above data suggested that S1PR_1/3_, but not S1PR_2_, was responsible for the angiogenic potential of ovarian cancer cells.

### Effect of SphK blockage on ovarian cancer angiogenesis *in vivo*

**Figure 6:**
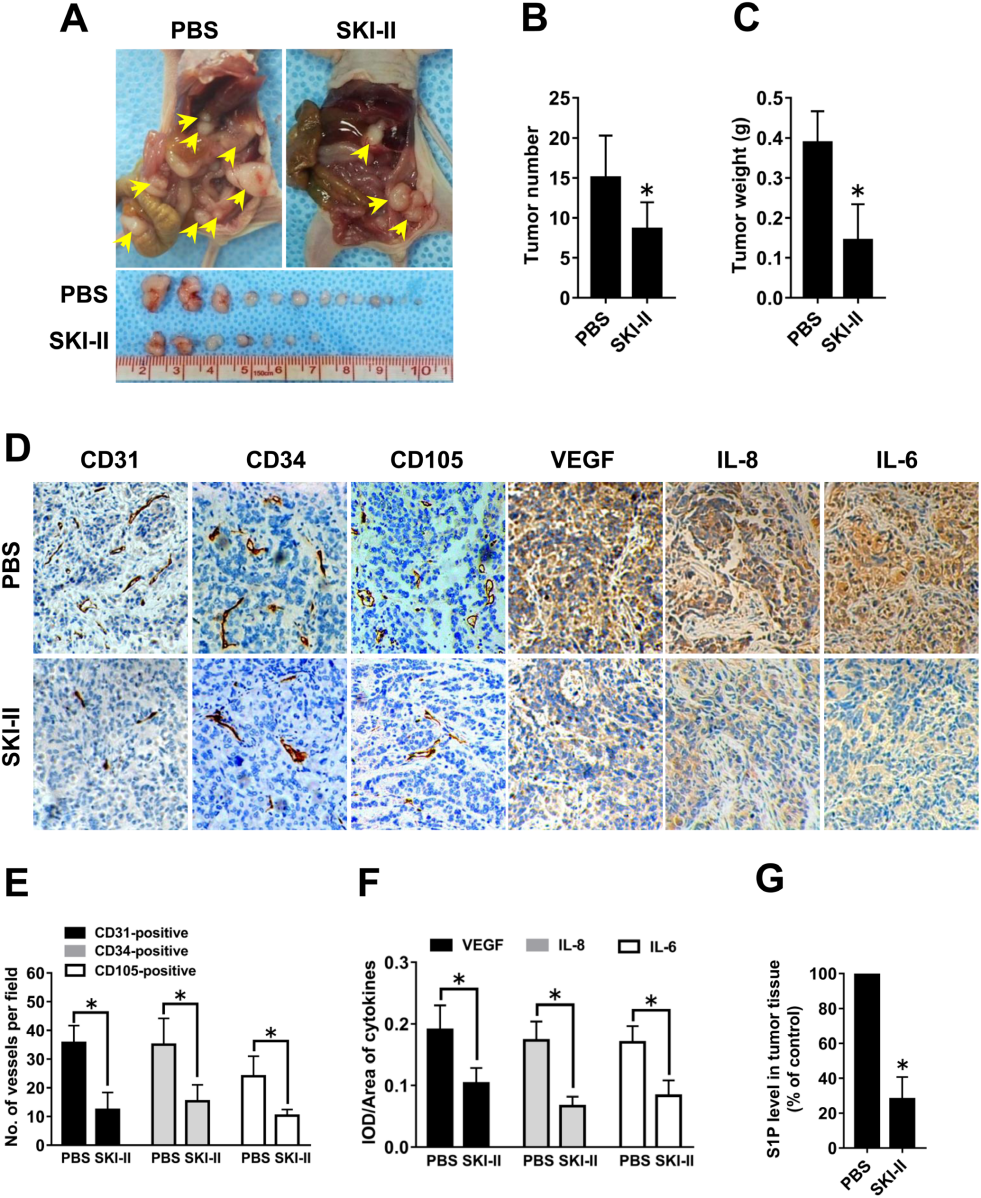
Effect of SphK blockage on angiogenesis *in vivo* **(A)** Representative images of disseminated tumors in intraperitoneal ovarian cancer xenograft model treated with PBS (n=8) or SKI-II (n=8). Tumor number **(B)** and tumor weight **(C)** were quantificated. **(D)** Immunohistochemical staining for CD31, CD34, CD105, VEGF, IL-8 and IL-6 was performed. **(E)** The number of CD31-positive, CD34-positive or CD105-positive vessels was quantificated. **(F)** Statistical analysis of integrated optical density (IOD)/area of VEGF, IL-8 and IL-6. **(G)** S1P levels in the tumor tissue were significantly deceased in the SKI-II treated group (* p<0.05 vs. PBS group).

In light of the *in vitro* data, we hypothesized that SphK blockage may also inhibit ovarian cancer angiogenesis *in vivo*. Ovarian cancer mouse models were intraperitoneally injected with SKI-II twice a week. SKI-II injection resulted in significantly decreased tumor number and tumor weight (Figure 6A-6C). Inspection of the tumors harvested from the experimental group revealed that tumors were smaller and less vascularized in the SKI-II group than those in PBS group (Figure 6A). The microvascular density (MVD) in the largest tumor of each mouse was characterized by immunohistochemical (IHC) staining using CD31, CD34 and CD105 antibodies that specifically recognize endothelial cells. The results revealed decreased MVD in the SKI-II group (Figure 6D-6E). The angiogenic factor expression levels in the tumor tissue were also characterized by IHC staining. The results showed that SKI-II administration significantly inhibited the VEGF, IL-8 and IL-6 levels in mouse models (Figure 6D and 6F). Moreover, the levels of S1P in tumor tissue were tested by ELISA. S1P levels were markedly decreased in the SKI-II treated group compared with the PBS group (Figure 6G). The above results indicated that the tumor growth, tumor angiogenesis, S1P and angiogenic factor expression of ovarian cancer could be blocked by SphK inhibitor *in vivo*.

### Effect of S1PR_1/3_ blockage on ovarian cancer angiogenesis *in vivo*

**Figure 7:**
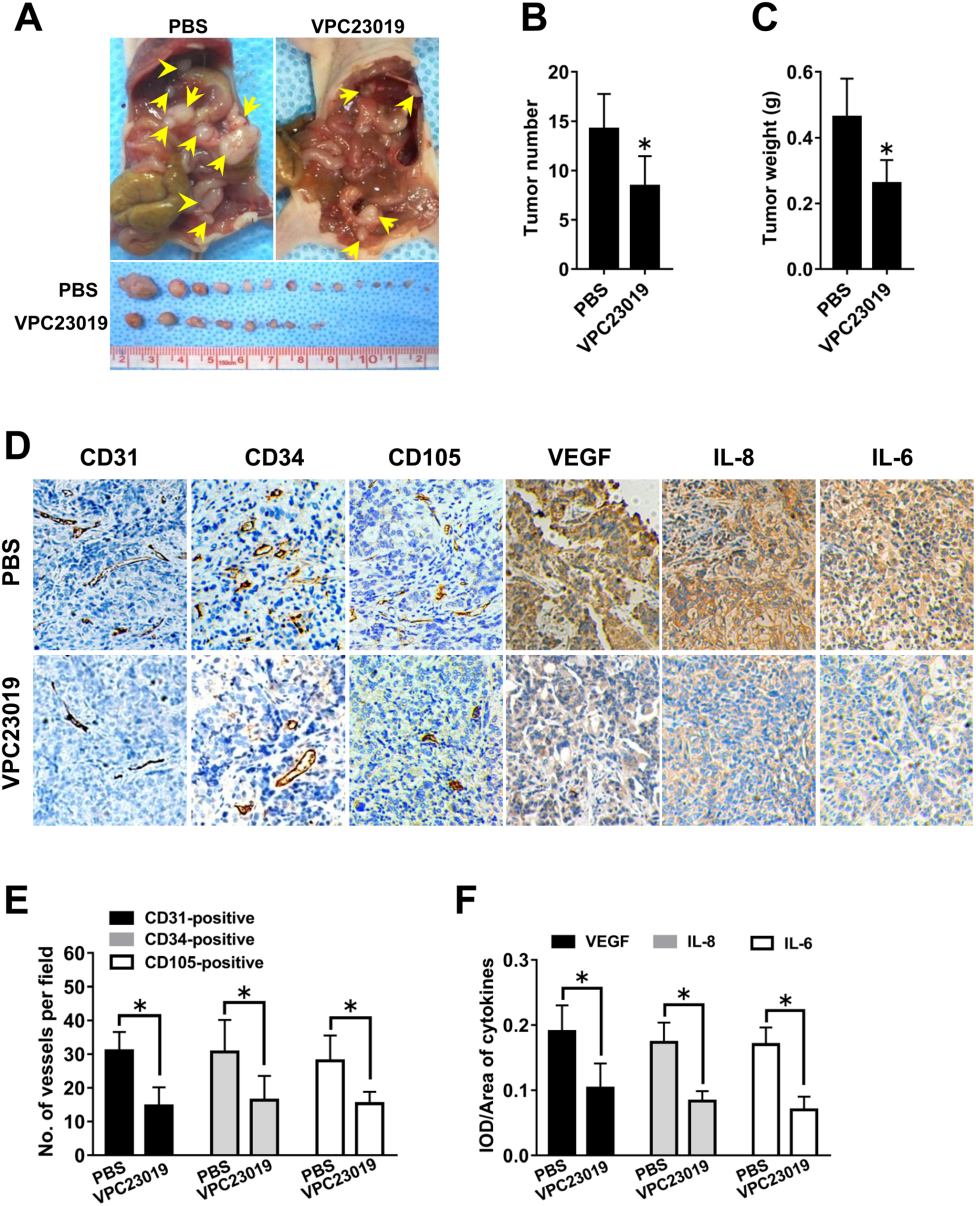
Effect of S1PR1/3 blockage on angiogenesis *in vivo* **(A)** Representative images of disseminated tumors in intraperitoneal ovarian cancer xenograft model treated with PBS (n=8) or VPC23019 (n=8). Tumor number **(B)** and tumor weight **(C)** were quantificated. **(D)** Immunohistochemical staining for CD31, CD34, CD105, VEGF, IL-8 and IL-6 was performed. **(E)** The number of CD31-positive, CD34-positive or CD105-positive vessels was quantificated. **(F)** Statistical analysis of IOD/area of VEGF, IL-8 and IL-6 (* p<0.05 vs. PBS group).

We found that S1PR_1_ and S1PR_3_, but not S1PR_2_, were responsible for the angiogenic potential and angiogenic factor secretion of ovarian cancer cells *in vitro*. Therefore, we further determined whether S1PR_1/3_ also regulated *in vivo* angiogenesis by intraperitoneally injecting the mouse models with S1PR_1/3_ antagonist VPC23019. We found that the tumor number and tumor weight were significantly inhibited by VPC23019 administration (Figure 7A-7C). Besides, the results of IHC showed that VPC23019 administration inhibited the MVD level and the angiogenic factor expression in mouse models. These results suggested that S1PR_1/3_ antagonist could block the tumor growth, tumor angiogenesis and angiogenic factor expression of ovarian cancer *in vivo*.

## DISCUSSION

In the current study, we provided evidence that SphK1/S1P/S1PR_1/3_ signaling played an important role in ovarian cancer angiogenesis and blockage of this pathway could significantly inhibit the angiogenic process of the cancer. Specially, our study provided the following new findings: (1) SphK1, but not SphK2, expression level was closely correlated with microvascular density (MVD) of ovarian cancer tissue; (2) The angiogenic potential and angiogenic factor secretion of ovarian cancer cells could be attenuated by SphK1, but not SphK2, blockage and were restored by the addition of S1P; (3) S1P induced the angiogenic factor expression via S1PR_1_ and S1PR_3_ in ovarian cancer cells; (4) Blockage of SphK or S1PR_1/3_ could effectively inhibit ovarian cancer angiogenesis.

SphK exhibits two isoforms, SphK1 and SphK2. SphK1 plays a critical role in ovarian cancer. Our previous study indicated that SphK1, elevated in ovarian cancer tissue, was involved in the metastasis of this deadly disease [9]. It was also reported that SphK1 promoted ovarian cancer cell proliferation and protected the cells from apoptosis through activating the survival pathways [16, 17]. However, the function of SphK2 in ovarian cancer remains largely unknown. Here, we explored the roles of SphK signaling in ovarian cancer angiogenesis, another important hallmark of this disease. As shown in Table 1, elevated level of SphK1 is accompanied by increased MVD in ovarian cancer tissue, which suggested the possible role of SphK in ovarian cancer angiogenesis. Therefore, we investigated the effect of SphK blockage on the angiogenic potential of ovarian cancer cells *in vitro*. As expected, SphK blockage by SKI-II, a specific inhibitor of SphK, significantly inhibited the angiogenic potential and the angiogenic factor secretion of the cells. In accordance with the *in vitro* results, SphK blockage also attenuated angiogenesis in a mouse ovarian cancer model. Moreover, SphK1, but not SphK2 knockdown, resulted in the suppression of the angiogenic potential and the angiogenic factor secretion of ovarian cancer cells, indicating that SphK1, but not SphK2, was responsible for ovarian cancer angiogenesis. These results suggested that SphK1 might be involved in ovarian cancer angiogenesis and raised the possibility that SphK1 might serve as a novel target to block tumor-associated angiogenesis in ovarian cancer.

The biological function of SphK mainly depends on its production of S1P, a bioactive sphingolipid generated from sphingosine by SphK [7]. We found that knockdown of SphK1, but not SphK2, by siRNA inhibited S1P secretion in ovarian cancer cells. Moreover, S1P expression could also be blocked by SKI-II in a mouse ovarian cancer model. S1P, known as a major proangiogenic factor, could stimulate endothelial cell proliferation, migration and capillary tube formation, which are the essential process of angiogenesis [18–20]. Besides endothelial cells, S1P has also been indicated to modulate many pathologic processes in ovarian cancer cells [21–23]. Notably, we found S1P pretreatment significantly promoted the angiogenic potential and the angiogenic factor secretion of ovarian cancer cells *in vitro* (Supplementary Figure 1), which suggested that the angiogenic effect of S1P also derived from its stimulation on cancer cells. S1P can act by binding to S1PRs. S1PRs, a group of G protein-coupled receptors, include S1PR_1_, S1PR_2_, S1PR_3_, S1PR_4_ and S1PR_5_ [24]. These five receptors are differentially expressed in various tissues and cells, leading to diverse functional outcomes. Normally, S1PR_1-3_ are widely expressed in almost all types of cells while S1PR_4-5_ are relatively restricted to the immune or nerve systems [25, 26]. We found S1PR_1-3_ were overexpressed in ovarian cancer tissues. To further assess the roles of these S1PRs in ovarian cancer angiogenesis, we used S1PR specific antagonists or siRNAs. Our results showed that down-regulation of S1PR_1_ or S1PR_3_, but not S1PR_2_, effectively inhibited S1P-induced VEGF, IL-8 and IL-6 secretion in ovarian cancer cells. These results indicated that S1P could induce the angiogenic factor secretion through S1PR_1_ and S1PR_3_. However, the exact mechanisms of the angiogenic factor secretion induced by S1P/S1PRs are still far from clear. For instance, besides S1PR_1-3_, the roles of other S1PRs in angiogenic factor secretion need further investigation. Moreover, S1P has been shown to promote the activation of several intracellular signal transduction cascades, including NF-κB signaling [27], ERK1/2 signaling [28] and Akt signaling [29]. Further studies need to be carried out to determine the signaling pathway through which S1P induced the angiogenic factor secretion. Importantly, S1P has been reported to elicit synergistic effects with other angiogenic cytokines, such as IL-8 and VEGF, to promote vascular network formation *in vitro* [30, 31]. Thus, the regulation of angiogenesis by SphK1/S1P/S1PR axis could be very effective, which may integrate the joint and interactive effects of multiple proangiogenic factors. In addition to VEGF, IL-8 and IL-6, there are also many other proangiogenic factors that participate in ovarian cancer angiogenesis [15, 32]. Further studies are needed in the future to evaluate the roles of the other proangiogenic cytokines in SphK1/S1P/S1PR axis. In view of the function of S1PR_1/3_ in S1P-induced angiogenic factor secretion, we further determined its roles in the angiogenic potential of ovarian cancer. We found that S1PR_1_ and S1PR_3_ blockage by VPC23019 also decreased the proangiogenic factor expression and attenuated angiogenesis *in vitro* and *in vivo*. Together, these results provided evidence that both S1P and S1PR_1/3_ are responsible for the angiogenic factor secretion and the angiogenic potential of ovarian cancer. Although the major biological effects of S1P are known to be mediated through S1PRs on the cell surface, some important roles of intracellular S1P have recently been discovered [33, 34]. Further studies are needed in the future to explore the functions of intracellular S1P in ovarian cancer angiogenesis.

Collectively, our findings presented evidence that the SphK1/S1P/S1PR_1/3_ axis played a critical role in regulating ovarian cancer angiogenesis. SphK1 could control the release of S1P, which was able to promote the secretion of some proangiogenic cytokines in ovarian cancer cells via S1PR_1_ and S1PR_3_. These cytokines, together with S1P, led to the tumor-associated angiogenesis and progression of ovarian cancer. The results showed a novel role of SphK1/S1P/S1PR_1/3_ axis within the ovarian cancer. As we know, bevacizumab, a monoclonal antibody targeting VEGF, is the first FDA-approved antiangiogenic agent for the therapy of recurrent ovarian cancer [35]. The success of bevacizumab has impelled us to search similar or more efficacious targets to block ovarian cancer angiogenesis. Since SphK1/S1P/S1PR_1/3_ axis regulated a set of important proangiogenic factors, this signaling may become a promising target for novel therapeutic approaches, which needs further evaluation.

## MATERIALS AND METHODS

### Reagents and antibodies

SphK inhibitor-2 (SKI-II) and Sphingosine 1-phosphate (S1P) were bought from Sigma-Aldrich. VPC23019 and JTE-013 was purchased from Santa Cruz Biotechnology. Calcein-AM was ordered from Dojindo Molecular Technologies. Antibodies against SphK1, SphK2, S1PR_1_, S1PR_2_, S1PR_3_, CD31, CD34, CD105, VEGF, IL-8 and IL-6 were ordered from Santa Cruz Biotechnology. Antibody against GAPDH was purchased from Sigma-Aldrich.

### Tissue samples

Tissue samples were collected from surgical patients, including 10 normal ovarian tissues and 50 primary epithelial ovarian cancer (EOC) tissues (stage I-II 24 cases, stage III-IV 26 cases). This study was approved by Institutional Review Board of Shanghai Jiaotong University. All patients provided informed consent.

### Cell lines and culture conditions

Human ovarian cancer cell lines (SKOV3 and HO8910PM) were bought from American Type Culture Collection (ATCC) and Cell Bank, Chinese Academy of Sciences. The human umbilical vein cell line (EA.hy926) was purchased from ATCC. These cells were cultured in DMEM complete media. When agonists or antagonists were used, cells were serum-starved overnight prior to treatment. Unless otherwise indicated, the drug-containing medium was replaced with the drug-free medium after the cells were pretreated for 12 hours.

### Mouse models

The animal studies were approved by the Institutional Animal Care and Use Committee of Shanghai Jiao Tong University School of Medicine. Female BALB/c nu/nu mice aged 6 weeks were ordered from the Chinese Academy of Sciences. To establish intraperitoneal transplantation models, 8 mice in each group were intraperitoneally injected with 5×10^6^ SKOV3 cells. 10 mg/kg b.w. SKI-II or 0.2 mg/kg b.w. VPC23019 was administered into mice twice per week starting on day 7 after the injection of SKOV3 cells. 30 days after injection of tumor cells, the mice were sacrificed and the weight and number of visible tumors were calculated.

### Immunohistochemistry

Formalin-fixed, paraffin-embedded specimens were used for immunostaining. Briefly, tumor sections were dewaxed and then rehydrated. The slides were heated near the boiling stage by microwave. After blocking, the slides were incubated with each primary antibody. Sections were then incubated with secondary antibody followed by treatment with 3, 3'-diaminobenzidine and counterstaining with hematoxylin. The intensity of immunostaining was scored as follows: strong (3), moderate (2), weak (1), or negative (0). The proportion of positively stained tumor cells was assessed as follows: no positive tumor cells (0), <25% (1), 26–50% (2), 50–75% (3), and >75% (4). Staining index (SI) was calculated as staining intensity score × proportion score. The protein expression level was considered to be high when score was >3, and low when score was ≤3. Software Image-Pro Plus 6.0 was also used to calculate the immunostaining intensity.

### Quantification of microvasculature density

Quantification of microvasculature density was performed as described before [36, 37]. Microvessels were identified by CD31, CD34 or CD105 staining. After immunostaining, the entire section was scanned at ×40 magnification to find the hot-spots, regions with the highest vascular density. The number of CD31-positive, CD34-positive or CD105-positive vessels were counted in hot spots at ×200 magnification, and then the mean value was calculated, which were then taken as the MVD_CD31_, MVD_CD34_ or MVD_CD105_. The mean MVD value of all the samples was used to classify samples in high or low MVD groups.

### Small interfering RNA (siRNA) and transient transfection

The chemically synthesized siRNAs targeting human SphK1 (5'-AAGAGCUGCAAGGCCUUGCCC-3'), SphK2 (5’-AACCUCAUCCAGACAGAACGA-3’) S1PR_1_ (5'-AAGCUACACAAAAAGCCUGGA-3'), S1PR_2_ (5'-AAUACCUUGCUCUCUGGCUCU-3'), S1PR_3_ (5'-CUGCCUGCACAAUCUCCCUTT-3') and the control siRNA (5'-AAUUCUCCGAACGUGUCACGU-3') were ordered from GenePharma. SiRNA transfection was performed by using Lipofectamine (Invitrogen). After 48 hours of transfection, the levels of the targeted genes were detected by Western blots.

### Cell migration assay

Transwell chambers (8 μm pore size, Corning, USA) were used to test the migration ability of the cells. The culture media (CM) were prepared from drug-pretreated ovarian cancer cells cultured in serum-free media for 24 h. Endothelial cells were re-suspended in the CM and placed into the upper chamber. The complete medium was used as a chemoattractant in the lower chamber. The migrated cells on the membrane were stained and counted after 8 h.

### Matrigel invasion assay

Transwell chambers, pre-coated with matrigel, were used to test the invasion ability of the cells. Endothelial cells, re-suspended in the indicated CM, were placed into the upper chamber. The complete medium was used as a chemoattractant. The invaded cells were counted after 24 h.

### Tube formation assay

Endothelial cells were re-suspended in the indicated CM and seeded on the Matrigel pre-coated plates to form tube like structures. After 8 hours, the cells were stained with 2 μM Calcein-AM. Tube like structures were observed and quantified by counting the number of connected tubes under ×100 magnification.

### Real-time RT-PCR

RNA was isolated by TRIzol Reagent (Invitrogen). SYBR Green RT-PCR was performed to measure mRNA levels, which were then calculated by using the 2^-ΔΔCt^ method. Primers were as follows: SphK1, 5′-CATTATGCTGGCTATGAGCAG-3′ (forward) and 5′-GTCCACATCAGCAATGAAGC-3′ (reverse); SphK2, 5′-GGTTGCTTCTATTGGTCAATCC-3′ (forward) and 5′-GTTCTGTCGTTCTGTCTGGATG-3′ (reverse); S1PR_1_, 5′-CCTCTTCCTGCTAATCAGCG-3′ (forward) and 5′-ACAGGTCTTCACCTTGCAGC-3′ (reverse); S1PR_2_, 5′-CATTGCCAAGGTCAAGCTGT-3′ (forward) and 5′-ACGATGGTGACCGTCTTGAG-3′ (reverse); S1PR_3_, 5′-TCAGCCTGTCTCCCACGGTC-3′ (forward) and 5′-ACGGCTGCTGGACTTCACCA-3′ (reverse); GAPDH, 5′-TGCACCACCAACTGCTTAGC-3′ (forward) and 5′-GGCATGGACTGTGGTCATGAG-3′ (reverse).

### Western blot analysis

Cells were harvested at 48 h post-transfection. Then we performed the western blotting as previously described [38].

### Enzyme-linked immunosorbent assay (ELISA)

Cell culture medium was collected after the indicated treatments. ELISA kits (R&D Systems) were used to determine the levels of VEGF-A, IL-8 and IL-6 according to the manufacturer’s instructions.

### S1P determination

As previously described [39, 40], the S1P level in the tumor tissue homogenate or the culture medium was analyzed using ELISA kits (Echelon Biosciences).

### Statistical analysis

Statistical analyses were performed using the SPSS software. MVD and SphK1/2 expression were examined using the Spearman correlation test. The values were presented as the mean ± SD and were analyzed by t-test or ANOVA (p<0.05 was considered significant).

## SUPPLEMENTARY MATERIALS FIGURE


