# Supplementary material for: Sphingosine kinase 1/sphingosine-1-phosphate (S1P)/S1P receptor axis is involved in ovarian cancer angiogenesis
Source: Oncotarget. 2017 Aug 24;8(43):74947–61. doi: 10.18632/oncotarget.20471 (PMC5650392; doi:10.18632/oncotarget.20471)
Supplement: Supplementary file 1 [file oncotarget-08-74947-s001.pdf]

Sphingosine kinase 1/sphingosine-1-phosphate (S1P)/S1P receptor axis is involved in ovarian cancer angiogenesis

SUPPLEMENTARY MATERIALS

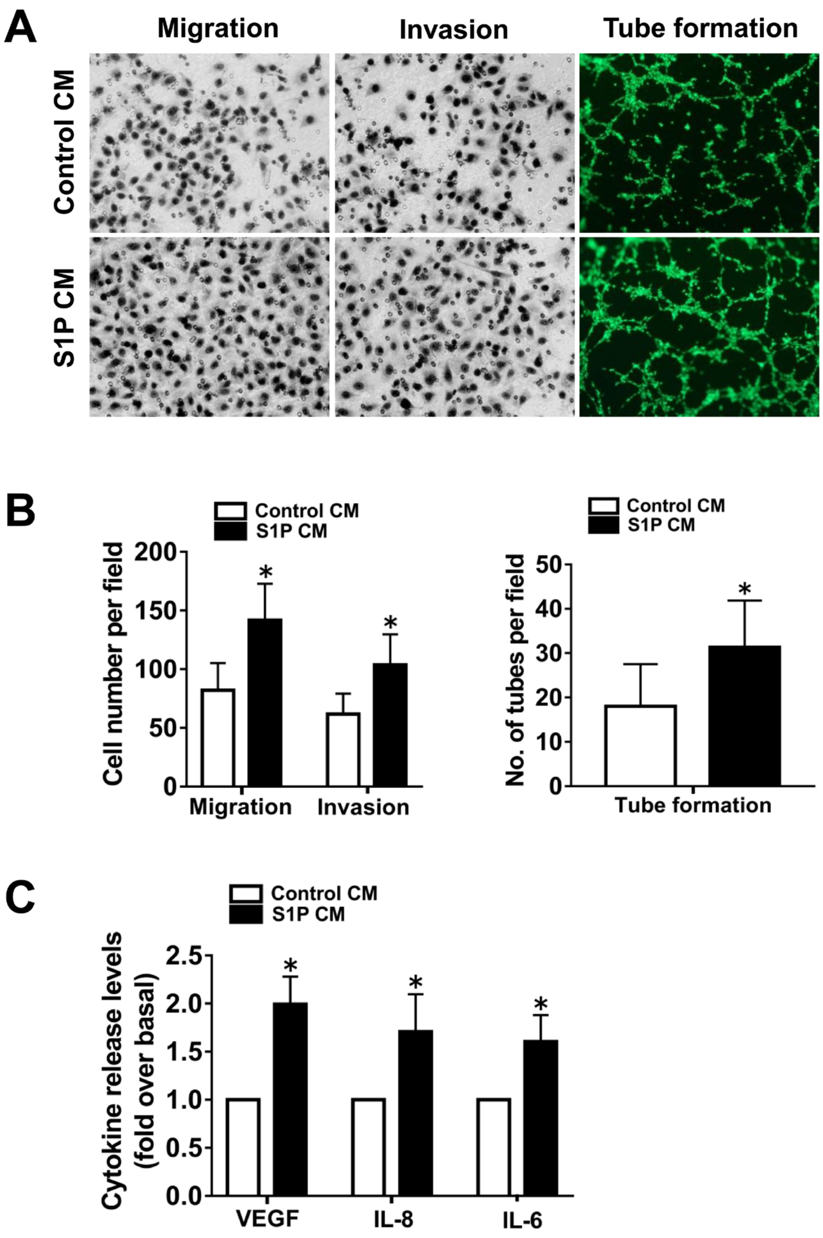

Supplementary Figure 1: Effect of S1P pretreatment on angiogenesis *in vitro*. (A) Representative images of the migration, invasion and tube formation assays. Endothelial cells were stimulated with CM from the ovarian cancer cells precultured with or without S1P (1μM). Migrated cells, invaded cells and tube like structures were photographed. **(B)** Statistical analysis of the migrated cells, invaded cells and tube like structures. **(C)** Effect of S1P pretreatment on the VEGF, IL-8 and IL-6 secretion of ovarian cancer cells. All experiments were repeated three times (*p<0.05 vs. Control group).
